# Bone morphogenetic protein-binding endothelial regulator of liver sinusoidal endothelial cells induces iron overload in a fatty liver mouse model

**DOI:** 10.1007/s00535-016-1237-6

**Published:** 2016-06-30

**Authors:** Takumu Hasebe, Hiroki Tanaka, Koji Sawada, Shunsuke Nakajima, Takaaki Ohtake, Mikihiro Fujiya, Yutaka Kohgo

**Affiliations:** 1 Division of Gastroenterology and Hematology/Oncology, Department of Medicine, Asahikawa Medical University, 2-1-1-1 Midorigaoka Higashi, Asahikawa, Hokkaido 078-8510 Japan; 2 Department of Legal Medicine, Asahikawa Medical University, Asahikawa, Japan; 3 Department of Gastroenterology, International University of Health and Welfare Hospital, Nasushiobara, Japan

**Keywords:** Non-alcoholic fatty liver disease, Iron metabolism, Signal regulation, Hepcidin, Whole-RNA sequencing

## Abstract

**Background:**

Non-alcoholic fatty liver disease (NAFLD) is frequently accompanied by iron overload. However, because of the complex hepcidin-regulating molecules, the molecular mechanism underlying iron overload remains unknown. To identify the key molecule involved in NAFLD-associated iron dysregulation, we performed whole-RNA sequencing on the livers of obese mice.

**Methods:**

Male C57BL/6 mice were fed a regular or high-fat diet for 16 or 48 weeks. Internal iron was evaluated by plasma iron, ferritin or hepatic iron content. Whole-RNA sequencing was performed by transcriptome analysis using semiconductor high-throughput sequencer. Mouse liver tissues or isolated hepatocytes and sinusoidal endothelial cells were used to assess the expression of iron-regulating molecules.

**Results:**

Mice fed a high-fat diet for 16 weeks showed excess iron accumulation. Longer exposure to a high-fat diet increased hepatic fibrosis and intrahepatic iron accumulation. A pathway analysis of the sequencing data showed that several inflammatory pathways, including bone morphogenetic protein (BMP)–SMAD signaling, were significantly affected. Sequencing analysis showed 2314 altered genes, including decreased mRNA expression of the hepcidin-coding gene *Hamp*. Hepcidin protein expression and SMAD phosphorylation, which induces *Hamp*, were found to be reduced. The expression of BMP-binding endothelial regulator (BMPER), which inhibits BMP–SMAD signaling by binding BMP extracellularly, was up-regulated in fatty livers. In addition, immunohistochemical and cell isolation analyses showed that BMPER was primarily expressed in the liver sinusoidal endothelial cells (LSECs) rather than hepatocytes.

**Conclusions:**

BMPER secretion by LSECs inhibits BMP–SMAD signaling in hepatocytes and further reduces hepcidin protein expression. These intrahepatic molecular interactions suggest a novel molecular basis of iron overload in NAFLD.

**Electronic supplementary material:**

The online version of this article (doi:10.1007/s00535-016-1237-6) contains supplementary material, which is available to authorized users.

## Introduction

Non-alcoholic fatty liver disease (NAFLD) is associated with liver steatosis caused by metabolic disorders without previous excessive alcohol intake. NAFLD includes non-alcoholic fatty liver (FL), which represents simple steatosis not associated with histological inflammation, and non-alcoholic steatohepatitis (NASH), which is associated with liver inflammation and/or fibrosis. The number of individuals with FL and NASH is increasing worldwide, particularly in the USA and in European and Asia-Pacific countries [1–3]. The number of obese people with a body mass index over 30 is also increasing, and this number includes more than 10 % of NASH patients. In a previous study, Tilg and Moschen suggested the multiple parallel hits hypothesis to demonstrate the exacerbation of NAFLD. This hypothesis posits that NAFLD pathogenesis has several causes, including oxidative stress caused by abnormal cytokine secretion by adipose tissue, iron overload or lipid peroxidation, and intestinal microbiota interactions with other genetic and epigenetic factors [4].

Iron overload is one cause of FL/NASH. Excess iron produces hydroxyl radicals, which subsequently oxidize lipids, proteins or nucleic acids, leading to hepatic fibrosis or tumorigenesis [5]. Alcoholic liver diseases and chronic hepatitis C frequently occur with iron overload, and can develop into cirrhosis and cancer [6]. Patients with NAFLD also show excessive iron levels, such as hyperferritinemia and hepatic iron accumulation. Accordingly, phlebotomy is often used to reduce serum alanine aminotransferase (ALT), and its effectiveness has been attributed to the reduction in free radicals [7, 8]. Iron overload pathogenesis is often due to dysregulation of the iron regulator hepcidin, which reduces iron absorption by degrading the iron transporters on intestinal epithelial cells and macrophages [9].

We and several other laboratories have reported that alcohol overload reduces hepcidin expression [10–12]. Downregulation of hepcidin via endoplasmic reticulum stress has been reported in a hepatitis C virus transgenic mouse model [13]. Hepcidin expression can be rescued in chronic hepatitis C patients who respond to interferon treatment [14]. Thus, we suggest that there is an important relationship between hepcidin reduction and chronic liver disease. However, the exact mechanism of iron overload in NAFLD is controversial. NASH patients have shown increased hepcidin expression concomitant with inflammation and intrahepatic iron deposition [15, 16]. In contrast, reduced hepcidin expression was found to increase iron absorption in patients with NAFLD [17].

In this study, we investigated iron overload in a high-fat diet-induced FL mouse model and examined the underlying mechanism by performing whole-RNA sequencing.

## Materials and methods

### High-fat diet-induced obesity in NAFLD mouse model

Eight-week-old male wild-type C57BL/6 mice (Charles River Japan Inc., Tokyo, Japan) were fed regular rodent diet (Oriental Yeast Company Ltd., Tokyo, Japan) or a lard-based high-fat diet (Oriental Yeast Company Ltd.). All mice were maintained under controlled conditions (22 °C, 50–60 % humidity, 12-h light/dark cycle) with food and water ad libitum. After 16 weeks (*n* = 10) or 48 weeks (*n* = 5) on the diets, mice were euthanized under anesthesia after 12-h starvation and were weighed, and blood was collected in heparin by cardiac puncture and centrifuged (15000×*g*, 5 min) for plasma collection. The livers were dissected and processed for histopathology, RNA sequencing, hepatic iron content, real-time quantitative polymerase chain reaction (RT-PCR) and western blotting. All experiments were performed in accordance with the rules and guidelines of the Animal Experiment Committee at Asahikawa Medical University.

### Plasma analysis

Mouse plasma concentrations of ALT and iron were measured using an automatic analyzer (Hitachi High-Technologies Corporation, Tokyo, Japan). Hepcidin concentration was analyzed by liquid chromatography/electrospray ionization–tandem mass spectrometry. Ferritin (ALPCO, Salem, NH, USA) and BMPER (USCN Life Science Inc., Wuhan, China) were analyzed by enzyme-linked immunosorbent assay (ELISA).

### Histopathological evaluation

Mouse livers were fixed in 10 % neutral-buffered formalin for 24 h at 4 °C and processed for paraffin embedding. The paraffin sections were prepared and subsequently dehydrated in graded ethanol and xylene. The sections were stained using hematoxylin and eosin (H&E), Masson’s trichrome or Berlin blue staining protocols and were immunostained with an anti-BMPER antibody (Abcam plc, Cambridge, UK).

### Hepatic iron content

Lipids were extracted from 100 mg liver tissue with 1 mL chloroform. Subsequently, the liver was desiccated using a micro-size centrifugal concentrator spin-dryer mini (TAITEC, Saitama, Japan), and was then weighed. The desiccated liver tissues were dissolved into concentrated nitric acid at 20 mg/mL. The solution was diluted with iron-free deionized water to bring the final nitric acid concentration to 0.1 mM. Non-heme iron was measured as hepatic iron content using an atomic absorption spectrophotometer (Hitachi, Tokyo, Japan) and expressed as μg non-heme iron/g dry liver tissue weight (μg Fe/g dry wt).

### RNA sequencing

Total RNA was isolated from the livers of mice fed the 16-week diets using a QIAGEN RNeasy Mini Kit (QIAGEN GmbH, Hilden, Germany). Ribosomal RNA was depleted using the RiboMinus Eukaryote System (Life Technologies, Carlsbad, CA, USA), and the remaining RNA was purified, fragmented, barcoded and reverse-transcribed using the Ion Total RNA-Seq Kit (Life Technologies). Sequencing was performed using Ion Torrent technology (Life Technologies), and whole-RNA expression analysis was performed using the Genomics Workbench system (CLC bio, Aarhus, Denmark). The expression of each gene was quantified as reads per kilobase of exon model per million mapped reads (RPKM). The average RPKM was calculated for the regular diet and high-fat diet groups, and their fold changes and *P* values were evaluated using Student’s *t* test. An absolute fold change of >1.5 and a *P* value > 0.05 were used to identify altered gene expression. Altered genes were further subjected to pathway analysis using MetaCore (GeneGo/Thomson Reuters, New York, NY, USA).

### RNA isolation and RT-PCR

Total RNA was isolated from the mouse livers using the QIAGEN RNeasy Mini Kit. RNA was reverse-transcribed by RETROscript using random decamers (Ambion/Life Technologies) to generate cDNA. Mouse *18S* rRNA was used as an endogenous amplification control to correct for variation in the efficiency of RNA extraction and reverse transcription. Specific primer pairs were used for *Hamp* mRNA, *Bmper* mRNA and *18S* rRNA (Life Technologies). The expression of *Hamp* mRNA, *Bmper* mRNA and *18S* rRNA in mouse livers was evaluated by RT-PCR (Life Technologies). All reactions were run in 96-well plates with a total volume of 20 μL. The reaction mixture consisted of 10 μL EagleTaq Universal Master Mix with ROX dye (Roche Diagnostics, Basel, Switzerland), 1 μL *18S* rRNA primer, 1 μL target primer, RNase free water and 250 μg sample cDNA. The PCR reaction involved the following steps: (1) 50 °C for 2 min to prevent carryover of DNA; (2) 95 °C for 10 min to activate polymerase; and (3) 40 cycles each of 95 °C for 15 s, 60 °C for 15 s and 72 °C for 45 s. The expression of *Hamp* and *Bmper* mRNA was analyzed using the comparative *C*_t_ method relative to *18S* rRNA expression.

### Western blot

Proteins were extracted from the mouse livers using RIPA buffer (150 mM NaCl, 0.25 % deoxycholic acid, 0.1 % sodium dodecyl sulfate [SDS], 50 mM Tris–HCl, pH 8.0). The protein concentration of each sample was determined by the Bradford method using the Pierce BCA Protein Assay Kit (Thermo Fisher Scientific, Waltham, MA, USA), according to manufacturer protocols. A total of 30 μg protein was subjected to electrophoresis using 12 % Mini PROTEAN TGX™ precast gels (Bio-Rad Laboratories, Hercules, CA, USA). The proteins were then transferred to nitrocellulose membranes (Bio-Rad), blocked in SuperBlock blocking buffer (Thermo Fisher Scientific) for 1 h at room temperature, and incubated with anti-SMAD1 rabbit antibody (Cell Signaling Technology [CST], Danvers, MA, USA), anti-phospho-SMAD1/5/8 rabbit antibody (CST), anti-β-actin mouse antibody (BD Biosciences), anti-BMPER antibody (Abcam) or anti-BMP6 antibody (Abcam) overnight at 4 °C. The membranes were incubated with horseradish peroxidase-conjugated anti-mouse or anti-rabbit IgG (R&D Systems, Minneapolis, MN, USA) and visualized using the SuperSignal West Pico Chemiluminescent Substrate (Thermo Fisher Scientific). The membranes were photographed with ImageQuant LAS 3000 (Fujifilm, Tokyo, Japan), and the band densities were analyzed using ImageJ software.

###  Immunoprecipitation (IP) of mouse plasma

Immunoprecipitation of mouse plasma was performed using Dynabeads (Life Technologies), as follows. Dynabeads were incubated with 2 μg of BMPER antibody (Abcam) or rabbit IgG (Abcam) for 1 h at room temperature. A total of 10 μL of plasma was precipitated for each sample. The samples were mixed with SDS buffer, and BMPER and BMP6 expression was analyzed by western blot.

### Primary hepatocyte and liver sinusoidal endothelial cell isolation from mouse livers

The livers from anesthetized C57BL/6 mice were perfused via the portal vein with Hanks’ balanced salt solution. After 10 min of perfusion, the liver was perfused for 10 min with Hanks’ solution containing 0.1 % collagenase (Wako, Osaka, Japan) suspended in phosphate-buffered saline (PBS). The cells were passed through a 100-μm cell strainer (Corning Life Sciences, Tokyo, Japan) and centrifuged for 3 min at 500 rpm. The pelleted cells were used as the hepatocyte-containing fraction. The hepatocyte fraction was washed three times with Williams’ E medium and cultured on a collagen-coated culture dish. The supernatant from the first centrifugation was used as the non-parenchymal cell-containing fraction. To isolate liver sinusoidal endothelial cells (LSECs), the cells were seeded on a collagen-coated dish in Dulbecco's modified Eagle's medium (DMEM; Wako), and after 1 h the dish was washed vigorously with DMEM to eliminate the other cells.

### Immunofluorescence

Isolated hepatocytes or LSECs were cultured on a coverslip (AGC Techno Glass Co., Shizuoka, Japan), fixed with paraformaldehyde for 5 min and blocked with SuperBlock blocking buffer (Thermo Fisher Scientific). The cells were incubated with an E-cadherin antibody (BD Biosciences, San Jose, CA, USA) and VE-cadherin antibody (Abcam) at 1:200 for 1 h at room temperature. Alexa Fluor 594 goat anti-mouse IgG and Alexa Fluor 488 goat anti-rabbit IgG (Life Technologies) were used as secondary antibodies for 1 h at room temperature with protection from the light. The cells were counterstained with DAPI and analyzed by fluorescence microscopy (Keyence Corporation, Osaka, Japan).

Mouse livers were mounted in Tissue-Tek O.C.T. compound (Sakura Finetek Japan Co Ltd., Tokyo, Japan) and frozen until preparation. Frozen sections were cut at 10-μm thickness and fixed in methanol for 10 min. Blocking was performed in PBS with 3 % bovine serum albumin (BSA). The slides were incubated with BMPER antibody (Abcam) at 1:200 in PBS overnight at 4 °C, and subsequently incubated with CD31 antibody (BD Pharmingen) at 1:100 in PBS for 1 h at room temperature. Alexa Fluor 594 goat anti-mouse IgG and Alexa Fluor 488 goat anti-rabbit IgG (Life Technologies) were incubated at 1:200 in PBS for 1 h at room temperature. The slides were counterstained with DAPI and analyzed using fluorescence microscopy.

### Statistical analysis

The graphs are expressed as scatter dot plots with a line representing the median. The RPKM results are expressed as the mean ± SD. Statistical significance was determined by Student’s *t* test with Welch’s correction. Statistical analyses were conducted using Prism version 5.01 statistical software (GraphPad Software Inc., La Jolla, CA, USA), and *P* < 0.05 was considered significant.

## Results

### Fatty liver, inflammation and iron overload in obese mice fed a high-fat diet

**Fig. 1:**
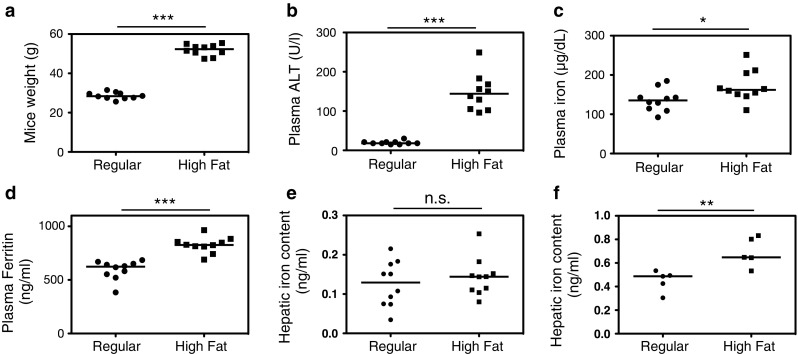
Fatty livers of obese mice and iron overload based on dietary treatment. Significant obesity was observed in the mice fed a high-fat diet (**a**). Elevated ALT levels (**b**) show the presence of fatty livers. Plasma iron (**c**) and ferritin (**d**) are increased in the mice fed a high-fat diet. Hepatic iron content was not significantly different after 16 weeks of a high-fat diet (**e**), but was significantly increased after 48 weeks of a high-fat diet (**f**). *P < 0.05, **P < 0.01, ***P < 0.001, *NS* not significant

**Fig. 2:**
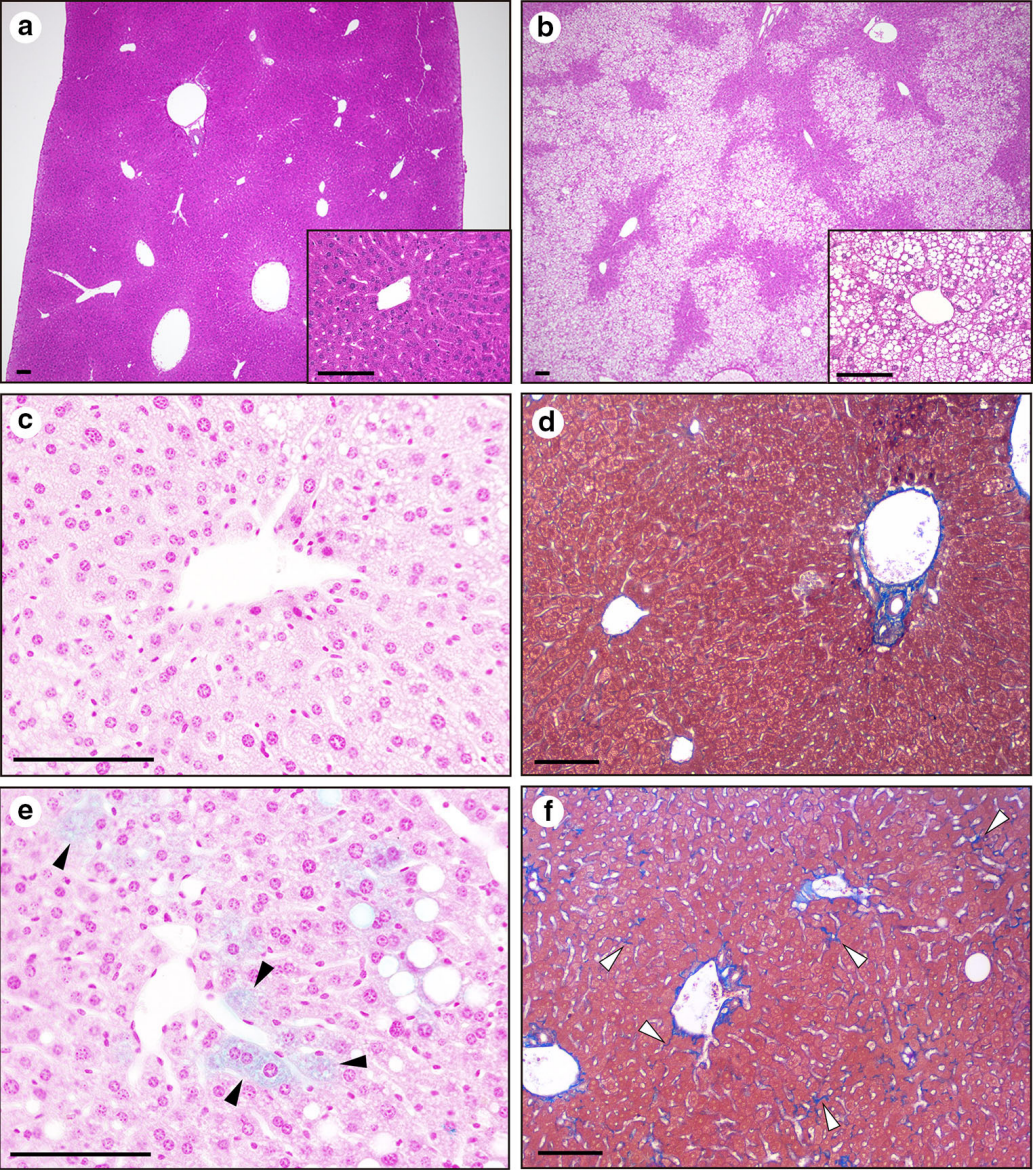
Histopathological status of mice fed a high-fat diet. The mice on a regular diet for 16 weeks had normal livers (**a**), whereas the livers of mice on a 16-week high-fat diet showed significant steatosis (**b**). Mice on a regular diet for 48 weeks did not exhibit iron accumulation (**c**) or fibrosis (**d**). Mice on a high-fat diet for 48 weeks showed iron accumulation with blue staining (**e** *black arrowhead*) and pericellular fibrosis (**f** *white arrowhead*). The *black bar* in each panel represents 100 μm

The body weight and plasma ALT were significantly higher in mice fed a high-fat diet for 16 weeks than in those fed a regular diet (Fig. 1a, b). Liver histology demonstrated significant hepatocyte steatosis, predominantly in the centrilobular zone, of the high-fat-diet mice (Fig. 2a, b). We did not observe hepatic fibrosis or iron accumulation in either group (data not shown). However, the livers of mice fed a high-fat diet for 48 weeks showed pericellular fibrosis and significant iron accumulation compared to the livers from mice on a regular diet (Fig. 2c–f). Therefore, 48 weeks of a high-fat diet induced NASH in mice. Plasma iron and ferritin were significantly higher in the mice fed a high-fat diet (Fig. 1c, d). The liver iron content was not significantly increased after 16 weeks on the high-fat diet (Fig. 1e). However, by 48 weeks, the mice fed a high-fat diet had increased liver iron content, whereas the mice fed a regular diet did not (Fig. 1f). These results suggest that at 16 weeks, the mice fed a high-fat diet exhibited hematological iron overload with little excessive hepatic iron, and further feeding of a high-fat diet caused iron accumulation in the liver, leading to hepatic inflammation and fibrosis that worsened NASH. To explore the underlying mechanism leading to NASH development, we focused on mice with FL after a 16-week high-fat diet for further analysis.

### RNA sequencing of obese mouse liver

**Fig. 3:**
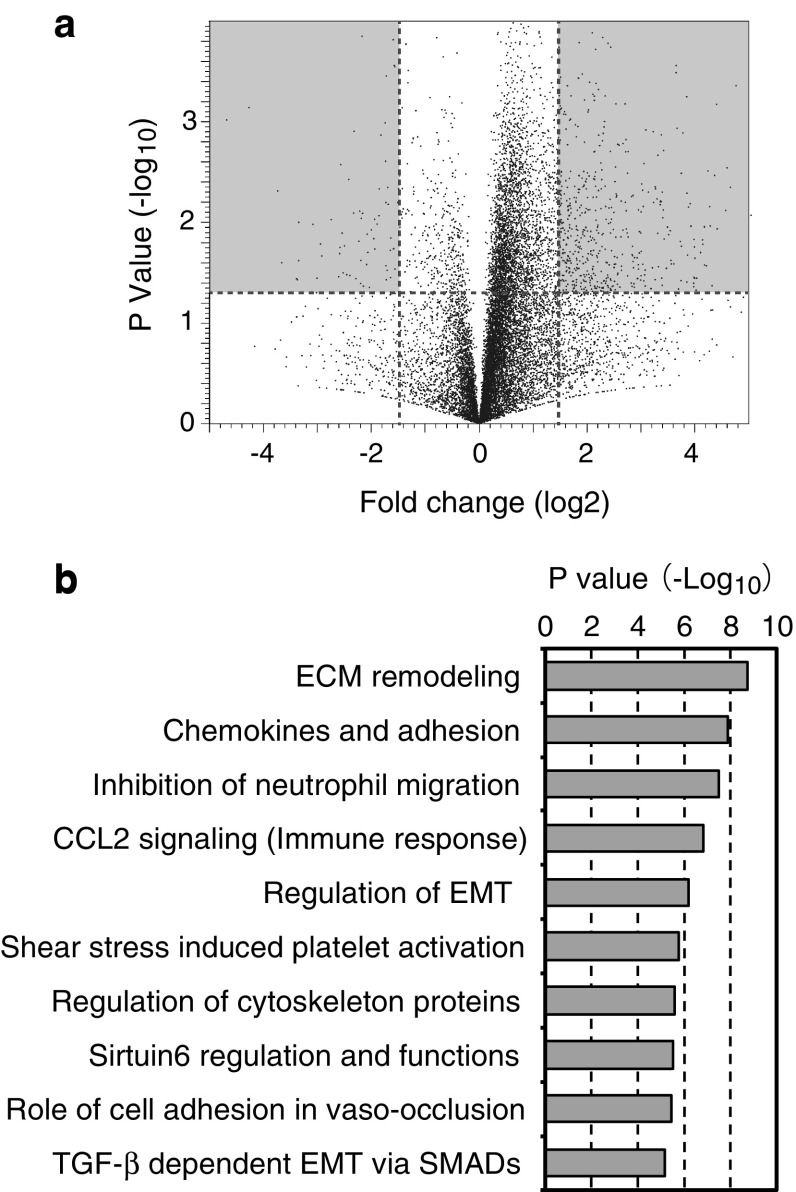
Gene expression analysis by RNA sequencing. **a** Volcano plot of the gene expression values The *gray area* represents 2314 genes whose expression was altered by a fold change >1.5 and *P* < 0.05. **b** The top 10 significant signals from the pathway analysis results

RNA sequencing was performed on the livers of mice fed a regular diet (*n* = 3) or a high-fat diet (*n* = 3) for 16 weeks. A total of 38,114 genes were analyzed, of which 2314 showed altered gene expression (Fig. 3a). To identify the primary molecular event in the liver tissue, we performed pathway analysis using MetaCore (Fig. 3b). The top 10 significant pathways showed several common characteristics, as follows. The “Sirtuin-6 regulation and functions” pathway showed that fatty acid synthase, HMG-CoA synthase, and LDHA and SREBP1 expression were increased, suggesting that the livers of mice fed the high-fat diet had increased fatty acid and glucose metabolism. The “Chemokines and adhesion,” “Inhibition of neutrophil migration,” “CCL2 signaling” and “Role of cell adhesion in vaso-occlusion” pathways showed common increased inflammatory signals, including TNF-α, CCL2, TLR2 and NF-κB. The “ECM remodeling,” “Regulation of EMT” and “TGF-β-dependent EMT via SMADs” pathways had common increased growth signals, including TGF-β2, TGF-β receptor and NOTCH-1. These results indicate mild inflammation in the fatty livers of this mouse model, but there was no significant hepatic inflammatory cell infiltration.

Because we identified several inflammatory signal pathways that activate SMADs and signal transducers and activators of transcription (STAT)-3, which both trigger hepcidin expression and further cause iron-deficiency in liver tissue, we speculated that most of the inflammatory pathways detected in our analysis affected SMAD and STAT3 transcriptional activity in fatty livers. Thus, we focused on the expression of 36 genes related to iron regulation (Table 1). Among these, we detected six genes with altered expression: *Bmp4*, *Bmper*, *Epor*, *Gdf15*, *Hamp* and *Hfe2*. These genes encode BMP4, bone morphogenetic protein (BMP)-binding endothelial regulator (BMPER), erythropoietin receptor, growth differentiation factor 15, hepcidin and hemojuvelin, respectively.

**Table 1:** Gene expression values of RNA sequencing

Gene	Fold change	*P* value^a^	RPKM^b^	
			Regular diet	High-fat diet
*Bmp2*	−1.41	0.0008	4.72 ± 0.14	3.34 ± 0.22
*Bmp4*	1.60	0.0322	0.88 ± 0.14	1.41 ± 0.24
*Bmp6*	1.04	0.7883	1.80 ± 0.35	1.87 ± 0.23
*Bmper*	5.24	0.0351	0.06 ± 0.07	0.36 ± 0.14
*Bmpr1a*	1.21	0.2182	2.70 ± 0.44	3.28 ± 0.52
*Bmpr2*	1.33	0.0070	8.23 ± 0.59	10.9 ± 0.70
*Cebpa*	−1.01	0.9542	147 ± 16.1	146 ± 23.0
*Chrd*	−1.20	0.5171	0.95 ± 0.21	0.79 ± 0.33
*Cp*	1.42	0.0151	47.5 ± 7.51	67.3 ± 3.72
*Epor*	2.30	0.0470	0.44 ± 0.10	1.03 ± 0.34
*Fam132b*	1.00	1.0000	0 ± 0	0 ± 0
*Fth1*	1.49	0.0078	941 ± 72.9	1404 ± 144
*Ftl1*	1.27	0.2227	1542 ± 166	1963 ± 476
*Furin*	1.07	0.3930	35.2 ± 3.40	37.6 ± 2.67
*Gdf15*	4.23	0.0150	1.68 ± 1.94	7.11 ± 1.23
*Hamp*	−3.32	0.0269	345 ± 122	104 ± 6.19
*Hamp2*	−1.42	0.2236	42.0 ± 13.0	29.5 ± 7.44
*Hfe*	1.23	0.0457	9.28 ± 1.23	11.4 ± 0.39
*Hfe2*	1.88	0.0108	17.7 ± 2.30	33.3 ± 5.54
*Hgf*	1.33	0.1261	3.70 ± 0.62	4.91 ± 0.88
*Il6*	1.00	1.0000	0 ± 0	0 ± 0
*Nog*	1.00	1.0000	0 ± 0	0 ± 0
*Raf1*	1.06	0.5705	8.27 ± 1.22	8.75 ± 0.56
*Slc11a2*	1.33	0.0597	3.47 ± 0.69	4.63 ± 0.31
*Slc40a1*	1.24	0.1212	25.7 ± 5.23	31.9 ± 1.54
*Smad1*	1.01	0.9363	3.26 ± 0.60	3.30 ± 0.44
*Smad2*	−1.17	0.4830	4.05 ± 1.00	3.47 ± 0.81
*Smad3*	1.21	0.2572	3.22 ± 0.51	3.90 ± 0.72
*Smad4*	1.10	0.1631	6.26 ± 0.26	6.90 ± 0.59
*Smad5*	1.29	0.0730	2.62 ± 0.45	3.40 ± 0.31
*Stat3*	1.24	0.0044	9.09 ± 0.61	11.2 ± 0.16
*Tfr2*	−1.01	0.7838	136 ± 7.02	134 ± 8.41
*Tfrc*	1.19	0.2501	1.68 ± 0.13	2.01 ± 0.40
*Tmprss6*	1.22	0.0063	92.9 ± 4.39	113. ± 5.24
*Trf*	1.21	0.1039	3589 ± 466	4334 ± 401
*Twsg1*	1.18	0.0554	9.15 ± 0.88	10.7 ± 0.57

^a^Statistical analysis was performed by *t* test with Welch’s correction

^b^Data represent the mean ± SD (*n* = 3)

### Hepcidin expression and regulation of SMAD phosphorylation are decreased in obese mice

**Fig. 4:**
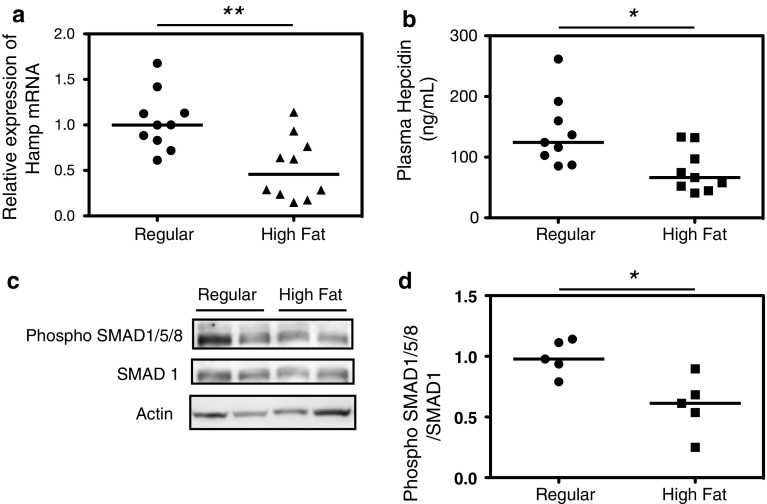
Hepcidin and phospho-SMAD expression in mice fed a high-fat diet. *Hamp* mRNA expression was significantly reduced in the livers of mice fed a high-fat diet (**a**). Hepcidin concentration in the plasma of the mice fed a high-fat diet was low (**b**). SMAD phosphorylation was reduced in the livers of mice fed a high-fat diet (**c**, **d**)

*Hamp* mRNA expression in mouse livers was significantly lower in the mice fed a high-fat diet for 16 weeks than in mice fed a regular diet (Fig. 4a). We measured the plasma hepcidin concentrations by liquid chromatography and found that they were also significantly lower in the mice fed a high-fat diet (Fig. 4b). To investigate the signals that regulate hepcidin expression, we focused on the BMP–SMAD pathway, because the RNA sequencing data identified three altered genes associated with this pathway (*Bmp4*, *Bmper* and *Hfe2*) and because the expression of *Bmper* showed the most dramatic fold change. Western blot analysis showed that phosphorylated SMAD1/5/8, which induces hepcidin transcription, was significantly decreased in the mice fed a high-fat diet (Fig. 4c, d).

### BMPER expression is increased in mice with FL and interacts with BMP6 in plasma

**Fig. 5:**
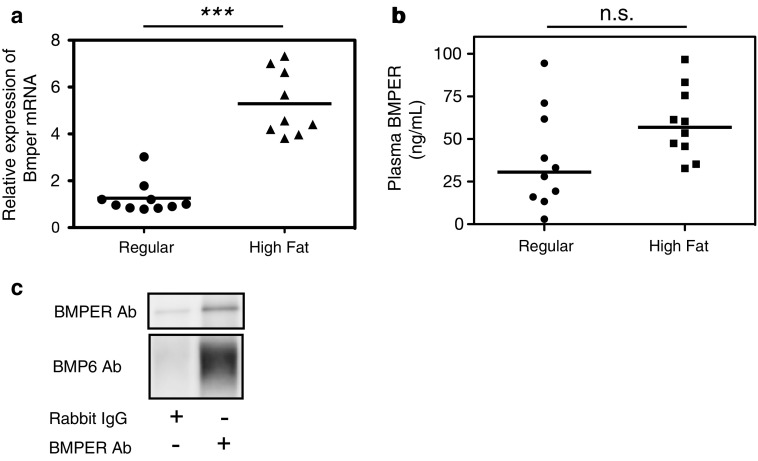
BMPER expression in mice fed a high-fat diet. **a** BMPER expression was high in the livers of mice fed a high-fat diet. **b** Plasma BMPER concentrations were not significantly different between the groups fed different diets, but there was an increasing trend in the mice fed a high-fat diet. **c** Immunoprecipitation showed specific BMPER precipitation and co-immunoprecipitation of BMPER with BMP6

Whole-RNA sequencing demonstrated that *Bmp4*, *Bmper* and *Hfe2* expression increased and SMAD phosphorylation decreased in the livers of mice fed a high-fat diet. BMPER is a known competitive inhibitor of BMP–SMAD signaling. Therefore, we focused on *Bmper* expression in the following experiments. RT-PCR of the mouse livers showed significant increases in *Bmper* mRNA in the mice fed a high-fat diet (Fig. 5a). The results of ELISA analysis of BMPER were not significant, but trended toward an increase in the mice fed a high-fat diet (Fig. 5b).

Western blotting of the IP plasma samples with the BMPER antibody showed the specific IP of BMPER from the plasma. BMPER co-precipitated BMP6, indicating that they bind to each other in plasma (Fig. 5c).

### BMPER is primarily expressed in LSECs rather than hepatocytes

**Fig. 6:**
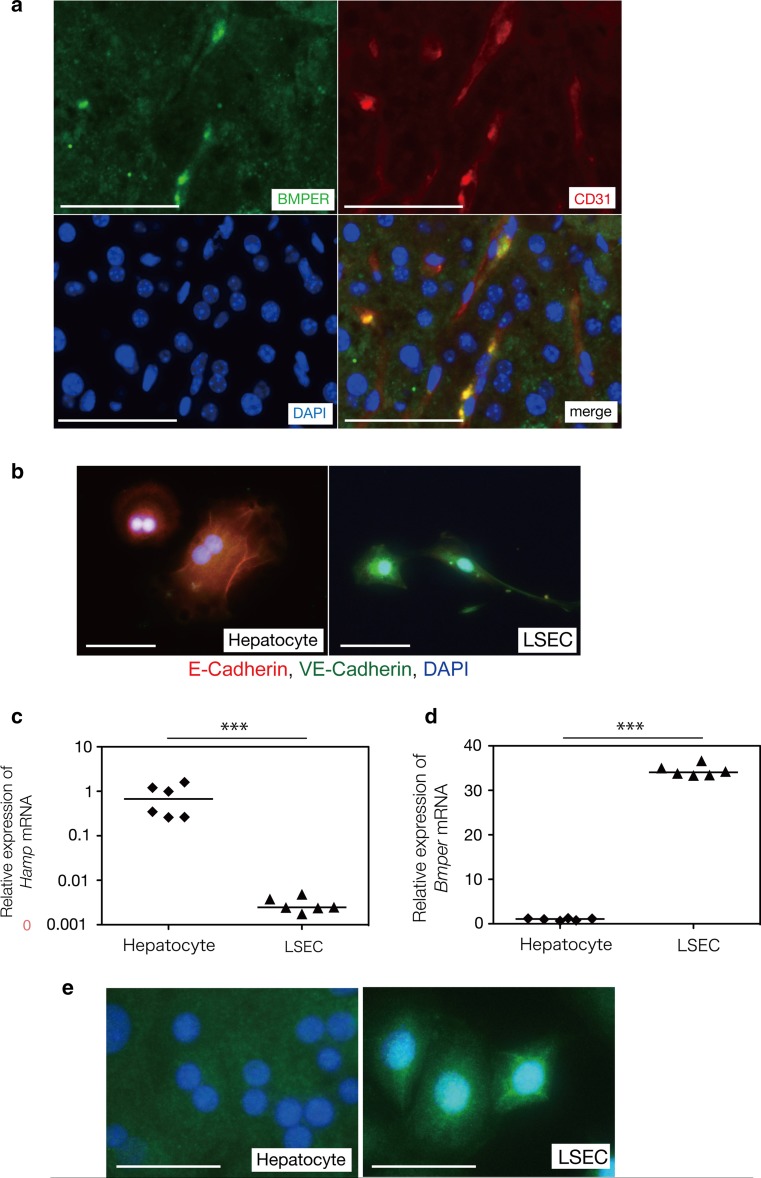
Liver sinusoidal endothelial cell-specific BMPER expression. Histological analysis showed that BMPER was expressed by CD31-positive endothelial cells (**a**). Specific cell isolation was confirmed by immunostaining (**b**). Hepatocytes showed higher expression of *Hamp* mRNA (**c**), and LSECs showed higher *Bmper* mRNA expression (**d**). BMPER immunostaining demonstrated that BMPER was strongly expressed in LSECs (**e**). The *white bar* inn each panel represents 50 μm

Immunostaining of the mouse livers with a BMPER antibody showed positive DAB staining in the sinusoid lumen (data not shown). We performed triple staining for BMPER, CD31 and DAPI on frozen sections of mouse livers. BMPER was expressed around the sinusoid lumen and overlapped with the expression of CD31, demonstrating that BMPER is expressed by LSECs (Fig. 6a). To confirm the population of BMPER-expressing cells, we isolated hepatocytes and LSECs from the mouse livers. We performed triple staining for E-cadherin, VE-cadherin and DAPI, confirming that the LSECs primarily expressed BMPER (Fig. 6b). We confirmed the pure isolation of cell populations by examining the expression of *Hamp* mRNA, which is specifically expressed in hepatocytes (Fig. 6c). *Bmper* mRNA expression in LSECs was 36-fold its expression in hepatocytes (Fig. 6d). The immunostaining for BMPER in hepatocytes and LSECs also demonstrated that BMPER was strongly expressed in LSECs (Fig. 6).

## Discussion

In this study, we showed that high-fat diet-induced FL coexisted with iron overload in mice due to the reduced expression of the iron regulator hepcidin. Hepcidin expression was suppressed by the increased expression of hepatic BMPER, which is primarily expressed by LSECs.

The plasma of FL mice showed iron overload and low hepcidin expression. Iron accumulation and ferritin levels have been reported to correlate with hepatic inflammation and fibrosis [17], which suggests that the high ALT and increased inflammatory signals detected by liver genome sequencing correlate with iron overloaded conditions. A pathway analysis of RNA sequencing data showed mild inflammation with increased TNF-α, TLR-2 and TGF-β signaling. These results suggest the presence of a small degree of inflammation in FL, which can worsen hepatitis even without inducing significant pathological inflammatory features. Hepcidin is a negative iron regulator secreted by hepatocytes, and its expression is induced by iron overload or inflammation and reduced by hypoxia or iron-deficient anemia [18]. The major signals regulating its expression are the BMP receptor–SMAD signal, the interleukin (IL)-6 receptor–STAT3 signal, and the transferrin receptor signal [19]. Hereditary hemochromatosis develops when hepcidin expression is reduced due to a mutated hepcidin regulatory signal [20–22]. Chronic hepatitis, such as chronic hepatitis C, and alcoholic liver diseases are often associated with the downregulation of hepcidin [11, 13]; however, hepcidin dysregulation in NAFLD remains controversial. Nelson et al. [17] reported that NAFLD iron overload was caused by hepcidin downregulation, whereas other reports have suggested that NASH patients have increased hepcidin expression due to inflammation [15, 16]. Although inflammation can increase hepcidin expression, our mouse model did not show strong inflammation, suggesting that reduced hepcidin expression is the dominant phenotype of FL.

Among the three major hepcidin-related signals, we focused on BMP–SMAD signaling and the regulator BMPER as a potential mechanism by which hepcidin is down-regulated. Another possibility is the IL-6–STAT3 pathway, which is an inflammatory signal. However, the FL mouse livers showed little evidence of inflammation, and genome sequencing detected no alteration in IL-6 expression in the mice fed a high-fat diet. Transferrin receptor signaling may induce hepcidin expression, but its signaling mechanism is not well known, and we did not observe a significant alteration in the expression of genes related to transferrin signaling by sequencing. The reduced expression of hepcidin and decreased SMAD phosphorylation in the livers of FL mice suggest that BMP signaling plays a significant role in FL. Because genome sequencing identified alterations in three genes related to BMP–SMAD signaling (*BMP4*, *BMPER*, *Hfe2*), we focused on the BMP signaling inhibitor BMPER. BMPER binds to BMP2, 4 and 6 to inhibit BMP signaling [23, 24]. Patel et al. reported that BMPER down-regulated hepcidin expression in genetically modified hypotransferrinemic mice [25]. There are two known forms of BMPER, soluble and membrane-associated, and the soluble form has known BMP inhibitory activity [26]. We showed that BMPER was increased in the serum under high-fat diet-induced FL conditions by assessing serum samples from FL mice. Other leptin-deficient ob/ob mice have shown FL and increased BMPER expression (data not shown), indicating that BMPER is increased in FL. We also showed that BMPER binds to BMP6. Therefore, BMPER inhibits hepcidin expression and thereby impairs iron metabolism.

The present study shows the importance of paracrine signaling in hepcidin regulation and the regulatory role of dominant BMPER expression by LSECs during this process. Previous reports have assumed that BMPER is expressed by hepatocytes [27]. BMPER can induce vascularization in the skin, heart and lung, and has been reported to be expressed by vascular endothelial cells [28], suggesting that hepatocytes could be a minor source of BMPER. To identify the BMPER-expressing cells in the liver, we isolated hepatocytes and LSECs, which revealed that BMPER was primarily expressed in the LSECs rather than hepatocytes. Enns et al. [29] reported that LSECs express the hepcidin regulator BMP6. Notably, we found that the hepcidin inhibitory molecule BMPER works as a paracrine molecule to regulate hepcidin expression.

There are some limitations to this study. We showed that iron deposition and NASH existed in mice fed a high-fat diet for 48 weeks. The plasma analysis of the 48-week NASH showed that hepcidin was decreased and BMPER was increased, but neither was significantly different (Figure S1). Some studies have shown that hepcidin was up-regulated in NASH [15, 16]; however, the mechanism is not clear. These variable hepcidin alterations in NASH could be caused by multiple factors, such as BMPER, inflammation, fibrosis and iron deposition. Further study is needed to clarify whether hepcidin and BMPER also interact in NASH conditions. We identified four additional hepcidin regulators (*Bmp4*, *Hfe2*, *Epor* and *Gdf15*) whose expression was altered in the mice fed a high-fat diet, but we did not study these regulators here. Although BMP6 is known to be a major regulator of hepcidin expression, BMP4 can also induce BMP–SMAD signaling in vitro [30]. Further studies are necessary to determine whether BMP4 induces hepcidin under NAFLD conditions. *Hfe2* is known to cause juvenile hemochromatosis, but its expression has been shown to be altered in NAFLD patients [31]. *Epor* and *Gdf15* are known to down-regulate hepcidin expression through hypoxia-related erythropoietin signaling [32, 33]. Thus, the mice in our study may have had hypoxia in the fatty liver, with steatosis occurring predominantly in the centrilobular zone. Moreover, we did not analyze other iron-associated organs, such as the fat, intestine, spleen and bone marrow. The molecular alteration of these organs should be determined in future studies.

In conclusion, we have shown that BMPER is a key molecule in iron overload under FL conditions, as it down-regulates hepcidin through paracrine mechanisms. Hepcidin is a known major regulator of iron metabolism expressed by hepatocytes; however, it is regulated by complex signal crosstalk. The regulation of hepcidin by hepatocytes and by LSECs is an important mechanism that warrants further study. As BMP–SMAD signaling primarily regulates hepcidin, BMPER is a novel molecule identified in this regulatory activity.

## Electronic supplementary material

Below is the link to the electronic supplementary material.

Figure S1. Hepcidin and BMPER expression in mice fed a high-fat diet for 48 weeks. The hepcidin concentration in the plasma of the mice fed a high-fat diet was low (a). The plasma BMPER concentration of the mice fed a high-fat diet was high (b). However, neither showed a significant difference. *n.s.* not significant (EPS 1126 kb)

## Abbreviations

NAFLD: Non-alcoholic fatty liver disease
BMP: Bone morphogenetic protein
IL: Interleukin
BMPER: Bone morphogenetic protein-binding endothelial regulator
LSEC: Liver sinusoidal endothelial cell
FL: Non-alcoholic fatty liver
NASH: Non-alcoholic steatohepatitis
ALT: Alanine aminotransferase
RT-PCR: Real-time quantitative polymerase chain reaction
ELISA: Enzyme-linked immunosorbent assay
RPKM: Reads per kilobase of exon model per million mapped reads
SDS: Sodium dodecyl sulfate
IP: Immunoprecipitation
STAT: Signal transducers and activators of transcription
